# Robust age estimation of southern sea otters from multiple morphometrics

**DOI:** 10.1002/ece3.6493

**Published:** 2020-07-12

**Authors:** Teri E. Nicholson, Karl A. Mayer, Michelle M. Staedler, Tyler O. Gagné, Michael J. Murray, Marissa A. Young, Joseph A. Tomoleoni, Martin Tim Tinker, Kyle S. Van Houtan

**Affiliations:** ^1^ Monterey Bay Aquarium Monterey CA USA; ^2^ U.S. Geological Survey Western Ecological Research Center Santa Cruz CA USA; ^3^ Department of Ecology and Evolutionary Biology Long Marine Laboratory University of California Santa Cruz CA USA; ^4^ Nicholas School of the Environment Duke University Durham NC USA

**Keywords:** aging, dentition, growth plate fusion, life history, marine mammals, tooth eruption, tooth wear

## Abstract

Reliable age estimation is an essential tool to assess the status of wildlife populations and inform successful management. Aging methods, however, are often limited by too few data, skewed demographic representation, and by single or uncertain morphometric relationships. In this study, we synthesize age estimates in southern sea otters *Enhydra lutris nereis* from 761 individuals across 34 years of study, using multiple noninvasive techniques and capturing all life stages from 0 to 17 years of age. From wild, stranded, and captive individuals, we describe tooth eruptions, tooth wear, body length, nose scarring, and pelage coloration across ontogeny and fit sex‐based growth functions to the data. Dental eruption schedules provided reliable and identifiable metrics spanning 0.3–9 months. Tooth wear was the most reliable predictor of age of individuals aged 1–15 years, which when combined with total length, explained >93% of observed age. Beyond age estimation, dental attrition also indicated the maximum lifespan of adult teeth is 13‒17 years, corresponding with previous estimates of life expectancy. Von Bertalanffy growth function model simulations of length at age gave consistent estimates of asymptotic lengths (male *L_oo_* = 126.0‒126.8 cm, female *L_oo_* = 115.3‒115.7 cm), biologically realistic gestation periods (*t*_0_ = 115 days, *SD* = 10.2), and somatic growth (male *k* = 1.8, *SD* = 0.1; female *k* = 2.1, *SD* = 0.1). Though exploratory, we describe how field radiographic imaging of epiphyseal plate development or fusions may improve aging of immature sea otters. Together, our results highlight the value of integrating information from multiple and diverse datasets to help resolve conservation problems.

## Graphical abstract

By synthesizing more than two decades of southern sea otter physical examination data from strandings, captive‐rearing for release, and field studies, we provide strong support for adopting minimally invasive aging techniques based on metrics obtainable during brief field capture. These techniques provide the means to gather age‐specific information from a living, wild population to inform management decisions focused on restoring sea otters and their ecosystems throughout California. Our results also highlight the value of integrating information from multiple and diverse datasets to help resolve conservation problems.

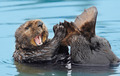

## 1 INTRODUCTION

Reliable aging methods are essential to develop and refine management strategies implemented to restore the southern sea otter *Enhydra lutris nereis* throughout California. After a century of federal and state protections following commercial exploitation, sea otters in California remain listed as “Threatened” under the U.S. Endangered Species Act of 1973. This protected status is primarily due to the population's small size (index = 2,962, Hatfield, Yee, Kenner, & Tomoleoni, 2019), vulnerability to a catastrophic oil spill, high mortality from infectious diseases (Kreuder et al., 2003; Miller et al., 2014) and shark bites (Moxley, Nicholson, Van Houtan, & Jorgensen, 2019; Tinker, Hatfield, Harris, & Ames, 2016), and significantly reduced geographic range (<15% of historical extent, USFWS, 2015). Given these threats, age‐explicit population data are essential to assess vital rates, identify vulnerable life stages, and inform recovery solutions. As keystone species in both kelp forests and eelgrass estuaries, sea otter recovery efforts may not only support their own population recovery (Mayer et al., 2019) but also bring cascading benefits for restoring these coastal ecosystems (Hughes et al., 2013, 2019; Kenner & Tinker, 2018).

Traditional sea otter aging methods rely primarily on tooth cementum analysis (Bodkin, Ames, Jameson, Johnson, & Matson, 1997; Schuler et al., 2018; Siniff & Ralls, 1988), but deposition and detection of discrete annual rings depends on seasonal environmental or physiological conditions (Matson, 1981). As a result, cementum annuli‐derived age estimation for southern sea otters is marginally accurate, successfully assigning age within 1 year error 35% of the time and within 2 years 50% of the time (CDFW, unpublished data). Beyond inaccuracy, this method requires tooth extraction, time‐consuming processing, and expensive and remote laboratory analyses, rendering it inadequate for aging this living, wild population (Mbizah, Steenkamp, & Groom, 2016).

In the absence of reliable aging methods, efforts to enhance southern sea otter age estimation should identify less invasive morphometrics that both capture age and maximize animal welfare. Typical phenotypic aging characteristics describe overall appearance in size, pelage, and tooth replacement or wearing; widely used for estimating age of carnivores (Chevallier, Gauthier, & Berteaux, 2017; Gay & Best, 1996; Gipson, Ballard, Nowak, & Mech, 2000; Lindeque & Skinner, 1984; Smuts, Anderson, & Austin, 1978; Stander, 1997; Van Horn, McElhinny, & Holekamp, 2003; Van Jaarsveld, Henschel, & Skinner, 1987). For sea otters, tooth wear, pelage characteristics, and body length are common field metrics for life stage estimation (Pattison, Harris, & Wendell, 1997), but their reliability in predicting specific or true age is less certain.

With technological advancements, detailed imaging of skeletal features during brief capture may enhance characterization of sea otter morphometry and improve aging accuracy. For example, veterinarians may use field radiographs to evaluate epiphyseal plate closure among appendicular long bones, refining age estimation between juvenile and adult stages. This technique is validated for aging Eurasian river otters *Lutra lutra* (Zeiler, 1988), cotton‐tail rabbits *Lepus sylvaticus* (Thomsen & Mortensen, 1946), raccoons *Procyon lotor* (Fiero & Verts, 1986; Sanderson, 1961), white‐tailed deer *Odocoileus virginianus* (Flinn, Strickland, Demarais, & Christiansen, 2013; Purdue, 1983), and black bears *Ursus americanus* (Marks & Erickson, 1966). As with other phenotypic traits, deriving and age‐validating the chronology of epiphyseal plate closure may further develop and refine more robust field aging methods.

We explore aging methods applicable to a living sea otter population by synthesizing more than two decades of physical examination data obtained during strandings (Nicholson et al., 2018), rearing for release (Mayer et al., 2019), and population monitoring (Tinker et al., 2006, 2013, 2017, 2018). These extensive, multi‐data sources enabled us to comprehensively examine ontological trends in morphology of known‐age individuals to evaluate standard field aging methods and identify noninvasive metrics that reliably predict age throughout a sea otter's lifespan. We also explore length at age relationships using Von Bertalanffy growth function (VBGF) models to estimate gestation (*−t*_0_), mean asymptotic length (*L_oo_*), and somatic growth (*k*). Finally, we consider the use of radiographs to refine aging metrics of young sea otters by defining timelines from epiphyseal fusion of limb long bones. By improving methods of aging from metrics obtainable during brief capture, this approach may enhance our ability to assess management strategies implemented to encourage range expansion and restore sea otters and their ecosystems throughout California.

## 2 MATERIALS AND METHODS

### 2.1 Sea otter captures and examinations

We physically examined sea otters during strandings, rehabilitation, and field studies throughout their mainland coastal range. Monterey Bay Aquarium (MBA) sea otter program staff and volunteers, in partnership with California Department of Fish and Wildlife (CDFW) and The Marine Mammal Center (TMMC), recovered stranded sea otter pups from Pigeon Point to Jalama Beach (1984–2018; 109 females, 129 males). Pups responding to treatment remained in captivity where staff examined and resampled individuals during rearing and weaning prior to release (2004–2018; 46 females, 19 males; e.g., Mayer et al., 2019). Field studies occurred along the Monterey Peninsula (1998–2017; 176 females, 55 males; e.g., Tinker, Doak, & Estes, 2008), Big Sur (2003, 2008–2011; 83 females, 16 males; e.g., Tinker et al., 2013), Piedras Blancas (2001–2003, 2012–2013; 72 females, 21 males; e.g., Tinker et al., 2006), Santa Barbara (2001–2002, 2012–2014; 21 females, 42 males; e.g., Tinker et al., 2017), and Elkhorn Slough (1998–2017; 28 females, 14 males; e.g., Mayer et al., 2019; Tinker et al., 2018). Although we examined the majority of wild sea otters during a single brief capture, nearly two decades of Monterey Peninsula and Elkhorn Slough sea otter studies permitted opportunistic resampling of known‐age individuals (aging error ≤1 year; 32 females, 14 males) during their lifespan.

### 2.2 Dental eruption timelines

By examining dentition of 38 stranded newborn pups (2004–2016; 25 females, 13 males; age ≤ 2 weeks) during rearing prior to release, we calculated first and full eruption timelines for all relevant teeth (8 deciduous and all 32 permanent). The remaining 18 of 26 deciduous teeth are either nonfunctional buds with short residence times (incisors 1 and 2), or present at birth (maxillary incisor 3, and all canines and second premolars). To identify newborns (1 day to 2 weeks), we referenced one or more of the following observations: a known birth event, presence and condition of umbilicus, traces of meconium in feces, and behavior (Payne & Jameson, 1984).

While pups were young, we conducted dental examinations weekly, but less frequently (monthly or quarterly) as pups matured and required greater restraint or chemical immobilization to reliably and safely examine. During dental examinations, we classified each tooth by ordinal eruption stage (absent, first eruption or emerging deciduous, partial deciduous, fully erupted deciduous, first eruption or emerging adult, partial adult, and fully erupted adult). Because we performed dental examinations at discrete intervals rather than continuously, we estimated age for each tooth when first and full eruption occurred as the midpoint between examinations directly before and after each stage was observed (Schuler et al., 2018). To maintain accuracy, we excluded individual eruption age estimates when examination intervals exceeded approximately 2 months.

### 2.3 Morphometric predictors of age

**FIGURE 1:**
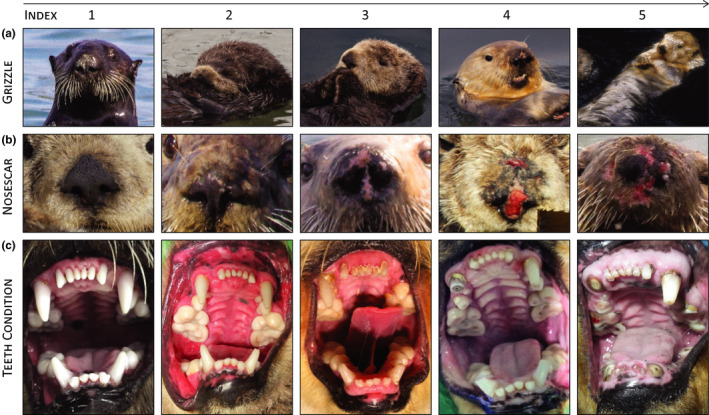
Common southern sea otter *Enhydra lutris nereis* field aging indices. (a) Pelage pigmentation loss (“grizzle”), (b) nose scarring, and (c) teeth condition or attrition are exemplified by five broad categories with grizzle defined as (1) none, (2) to eyes, (3) to lambdoidal crest, (4) to chest, and (5) to tail; nose scarring scaled as (1) none or black, (2) slight or white, (3) moderate or pink, (4) severe or red, and (5) extreme or significant tissue damage and loss; and teeth condition or attrition scored as (1) no signs of wear, canines entire and pointed, teeth often bright white, (2) light wear with canines slightly rounded but molar cusps prominent, (3) moderate wear with rounded or broken canines, caries present, flattened molar cusps, and one or more missing teeth, (4) severe wear or teeth worn nearly to gums, extensive caries, broken and missing teeth, and (5) extreme or most teeth worn to gumline or fractured

During field examinations of wild sea otters, we recorded a standard set of morphometrics and life history indices. Although information obtained for each individual varied by sample, the complete set included weight, total length (straight dorsal, nose to tail tip), xiphoid girth (transverse circumference at sternal process), tail length, paw width, baculum length (males only), canine width, pelage pigmentation loss (“grizzle”), nose scarring from repeated mating events (females only) or fighting (typically males), and teeth condition. We characterized grizzle by traditional rating scale: (1) none, (2) to eyes, (3) to lambdoidal crest, (4) to chest, and (5) to tail (Figure 1a; e.g., Pattison et al., 1997); and degree of nose scarring by defined index: (1) none or black, (2) slight or white, (3) moderate or pink, (4) severe or red, and (5) extreme or significant tissue damage and loss (Figure 1b). We described teeth condition by overall dental attrition from wearing, pitting, and fracturing, coded as (1) none, (2) slight, (3) moderate, (4) severe, and (5) extreme (Figure 1c, e.g., Pattison et al., 1997). When two consecutive codes reasonably described detailed aspects of grizzle, nose scarring, or teeth condition, we assigned half increment or mean values (e.g., 1.5, 2.5, 3.5, or 4.5) for refinement.

To evaluate field age estimates and develop a reliable model for aging southern sea otters, we first screened all morphometrics and indices by calculating correlation coefficients between each pairing (Peterson et al., 2018). For highly correlated pairings, we excluded metrics most sensitive to variation from factors other than age (e.g., seasonality or pregnancy, and measurement or recorder error) or less frequently available. We then used general (log‐) linear models (R Core Team, 2019) to test which of the remaining metrics best predicted female known age (*n* = 32), selecting our final model based on lowest AIC (Burnham & Anderson, 2002; Mazerolle, 2019). With these results, we derived a similar model from a limited sample of known‐age wild males (*n* = 14) and examined the relative importance of each models' metrics (Gromping, 2006). To assess model performance, we used repeated k‐fold cross‐validation (Kuhn, 2008), estimating prediction errors when applied to novel data subsets. Finally, we evaluate standard aging methods from population studies by comparing model and field age estimates (Tinker et al., 2018) from a sample of 219 wild sea otters (160 females, 59 males, 2006–2017) throughout California.

### 2.4 Tooth eruption, replacement, and attrition

To examine age‐related trends in broad functional tooth categories from eruption to attrition and loss, we combined tooth eruption and replacement patterns from our 38 stranded newborns with dental examination details from wild sea otters (*n* = 219). During dental examinations of individuals older than juvenile stage (age ≥ 1 year), we classified each tooth by attrition category (wearing, pitting, and fracturing) and intensity (none, mild, moderate, or severe). We then pooled the data by tooth type and age, calculating the proportion of total molars, premolars, canines, and incisors within each of six eruption stages (absent, emerging deciduous, partial or full deciduous, emerging adult, partial adult, and full adult) by 2‐week age increments to year 1, or by severity of each attrition category for every year of age 1 or greater (1–15). Ages for all sea otters were derived from either known‐age individuals, or field age estimates ground truthed from aging model results.

### 2.5 Von Bertalanffy growth functions

Tangential to aging, we explored population growth parameters by fitting a Von Bertalanffy growth function (VBGF) to an extensive sample of sea otter length at age data:

(1) $$L\left(t\right)=L_{\mathrm{oo}}\left(1-e^{-k\left(t-t_{0}\right)}\right)$$

where *L*(*t*) is the predicted length at age *t*, *L_oo_* is the population mean asymptotic length, *k* is a growth parameter of dimension time^−1^, and *t*_0_ is the age at length = 0, or (‐) gestation. The VBGF is a robust tool for modeling age to size relationships (i.e., mass, length, and girth) across multiple taxa of marine vertebrates (Estess et al., 2014; Garde, Heide‐Jørgensen, Hansen, Nachman, & Forchhammer, 2007; Nielsen et al., 2016; Van Houtan, Andrews, Jones, Murakawa, & Hagemann, 2016), including sea otters (Laidre et al., 2006; Palomares & Pauly, 2008; Tinker et al., 2013, 2017).

Because field captures resulted in measurements of sea otters primarily juvenile stage and older (94.2%, *n* = 493), we supplemented our dataset with total length at age records from 238 stranded pups to adequately characterize developmental stages from infancy to weaning and improve model reliability during early growth. These strandings occurred throughout the California range from 1984 to 2017, and demographics were evenly balanced among very small (33.6%, *n* = 80 age < 3 weeks), small (38.7%, *n* = 92, age 3–9 weeks), and large (27.7%, *n* = 66, age ≥ 10 weeks) pups. We estimated stranded pup age by tooth emergence and replacement patterns, and used either field age estimates or known‐age for all individuals captured during population studies. For each age estimate, we also defined an aging error, ranging from <1 day (for a known birth event) to 3 years (for individuals first examined as aged adults). A (±) 3‐year maximum error is consistent with other studies quantifying the precision of aging carnivores from tooth wear only (Chevallier et al., 2017; Galbany et al., 2018; Gipson et al., 2000).

During each model run (*n* = 10,000), a single length was drawn for every wild sea otter from population studies (*n* = 523). To construct a representative sample of lengths from pup strandings (*n* = 238), we randomly selected an age‐stratified subset (*n* = 28, age 2 days to 16 weeks), reducing potential size biases related to premature birth or weaning. Age was then assigned from an even distribution within a range defined by the otter's estimated age ± assessed aging error. A Levenberg‐Marquadt nonlinear least squares algorithm approximated parameters for Equation (1) (Elzhov, Mullen, Spiess, & Bolker, 2016). Because species' gestational period is generally fixed and independent of gender (Racey, 1981), we estimated *t*_0_ by pooling data from both sexes, but then calculated estimates of *k* and *L_oo_* on sex‐specific splits of the full dataset.

### 2.6 Bone growth plate closures

From 2013 to 2018, we recorded 204 total radiographs of 37 individual sea otters (26 females, and 11 males) that stranded as pups. From these radiographs, we examined 11 long‐bone growth plates (proximal and distal humerus, radius, and tibia; proximal fibula; distal ulna and femur; and olecranon and tibial tuberosity), assigning each to one of two categories: either open or closed. Radiographs of closed or completely fused plates were characterized by a uniform cortex, uninterrupted by radiolucent cartilage (Smith, 1968). We then approximated fusion timelines for each epiphyseal plate using binomial logistic regression (R Core Team, 2019) to predict probability of closure by age. Age assignment was based on examination of pup's tooth eruption and replacement patterns at stranding. After stranding, radiography frequency for each pup was variable (median = 4, range 1–19), coinciding with routine physical examinations, tagging, or instrumentation in preparation for release. We recaptured five females after release, either housing temporarily or permanently, extending their radiography samples to adulthood.

We produced all graphical figures in R (R Core Team, 2019), using the package ggplot2 (Wickham, 2016).

## 3 RESULTS

**FIGURE 2:**
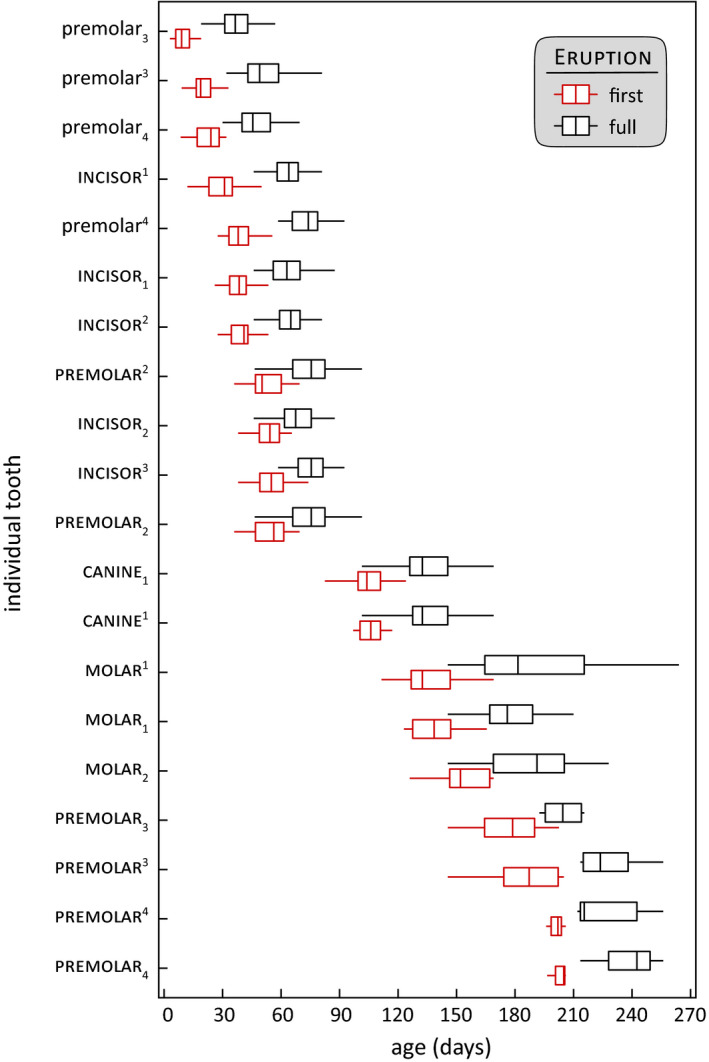
Detailed tooth eruption timelines are a reliable means for aging juvenile sea otters. Box plots (with quartiles and the interquartile range) chronicle the age ranges for the first eruption (red) and full emergence (black) of four deciduous (lower case) and all permanent (capitalized) teeth. Superscript and subscript indicate maxillary or mandibular, respectively. Numbers define sequential position. Data are derived from 38 individuals (25 females and 13 males) who stranded as neonate pups, ≤14 days old. The remaining deciduous teeth we do not describe as they either have a short residence time or are already present at birth. This chart spans 0.3–9 months of age and can serve as a rigorous benchmark to estimate the age of first year sea otters. For each tooth, box plots are jittered on the *y*‐axis for visualization. Because we did not detect differences related to gender, data for males and females are pooled

Deciduous and permanent tooth first and full eruption schedules span from a pup's second week midway through its juvenile stage at approximately 9 months of age, with identifiable metrics during every month of development (Figure 2, Tables A1 and A2). Premolars 3 and 4 are the only functional deciduous teeth absent at birth, and first erupt in sequential order, mandibular set before maxillary, between 9 and 38 days. The first permanent teeth to erupt are the incisors, in sequence but maxillary before mandibular, between 31 and 55 days. All permanent second premolars first erupt between 50 and 56 days, with the final two incisors, mandibular incisor 2 and maxillary incisor 3. All permanent canines first erupt together between 104 and 106 days, followed by permanent molars in sequential order between 133 and 152 days, and remaining premolars (3 and 4) sequentially between 179 and 205 days. Full eruption of each individual tooth when measured from its first eruption occurred in approximately 30 days. We did not detect significant differences in eruption timelines between males and females (*t* = 0.72, *p* = .47, *n* = 18), or right and left sides of the jaw (*t* = 1.29, *p* = .21, *n* = 18), so we pooled data based on these factors.

**FIGURE B1:**
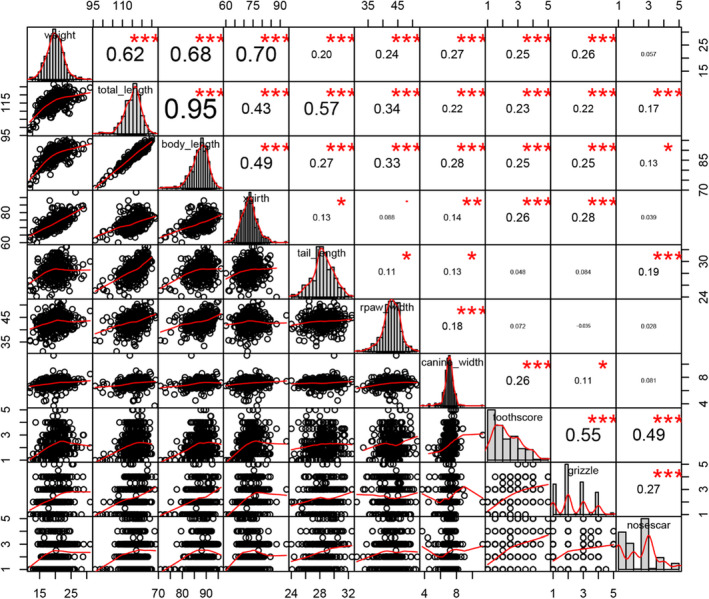
Correlation matrix (Peterson et al., 2018) of wild female sea otter (*n* = 345) morphometrics and life stage indices. Morphometrics examined are weight (kg), total length (cm, straight dorsal measure from nose to tail tip), body length (cm, total length minus tail length), xgirth (cm, transverse circumference at xiphoid or sternal process), tail length (cm), rpaw width (cm, right paw width), and canine width (mm). Life stage indices are tooth score, or degree of overall dental attrition from wearing, pitting, and fracturing as (1) none, (2) slight, (3) moderate, (4) severe, and (5) extreme; grizzle or pelage pigmentation loss as (1) none, (2) to eyes, (3) to lambdoidal crest, (4) to chest, and (5) to tail; and nose scarring as (1) none or black, (2) slight or white (3) moderate or pink, (4) severe or red, and (5) extreme or significant tissue damage and loss

**FIGURE B2:**
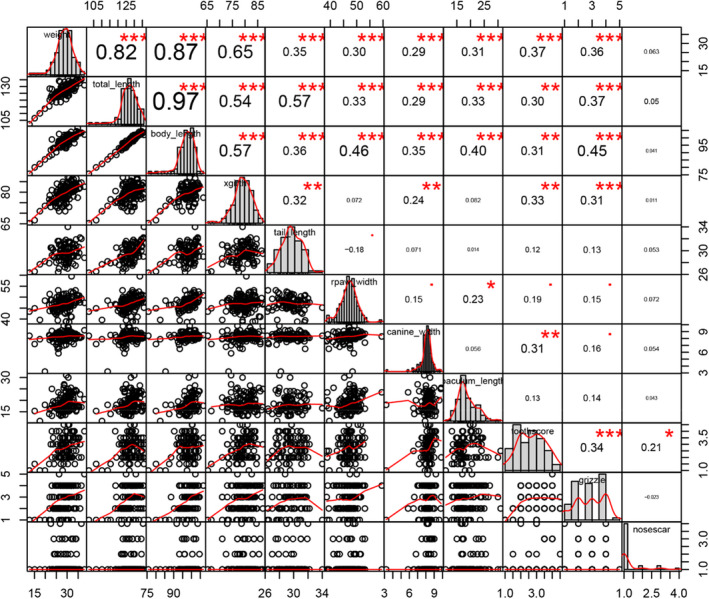
Correlation matrix (Peterson et al., 2018) of wild male sea otter (*n* = 123) morphometrics and life stage indices. Morphometrics examined are weight (kg), total length (cm, straight dorsal measure from nose to tail tip), body length (cm, total length minus tail length), xgirth (cm, transverse circumference at xiphoid or sternal process), tail length (cm), rpaw width (cm, right paw width), canine width (mm), and baculum length (cm). Life stage indices are tooth score, or degree of overall dental attrition from wearing, pitting, and fracturing as (1) none, (2) slight, (3) moderate, (4) severe, and (5) extreme; grizzle or pelage pigmentation loss as (1) none, (2) to eyes, (3) to lambdoidal crest, (4) to chest, and (5) to tail; and nose scarring as (1) none or black, (2) slight or white, (3) moderate or pink, (4) severe or red, and (5) extreme or significant tissue damage and loss

When screening phenotypic traits to build a model for aging sea otters 1 year or older, several size‐related morphometrics among individuals from population studies were highly correlated, including weight, total length, body length, and girth (Figures B1 and B2), so we disqualified all except for total length, because this measurement was most frequently available and less likely than weight or girth to be affected by short‐term fluctuations or cycles in prey availability, nutrition, or reproductive status (i.e., pregnancy). Our remaining metrics included total length, tail length, paw width, canine width, and life stage indices for grizzle, nose scar, and overall teeth condition.

**FIGURE 3:**
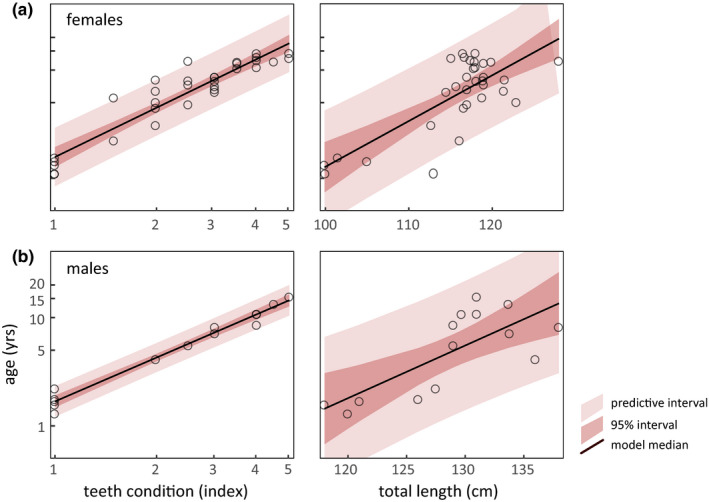
Model morphological indices of sea otters as they relate to age. (a) Teeth condition and (b) total length were the most reliable predictors of known age among wild female and male sea otters, across ages 1–15 years. Teeth condition index describes overall degree of wearing, pitting, and fractures, where 1–5 symbolizes none, slight, moderate, severe, and extreme attrition, respectively. Total length is the straight dorsal measurement from the outer tip of the nose to tail tip. The log–linear relationship of age to each metric is described by a model (black line, with confidence intervals) where open circles are individual sea otters (females = 32 and males = 14). Of all available field metrics, teeth condition (a) is the most accurate means to estimate wild sea otter age

**FIGURE B3:**
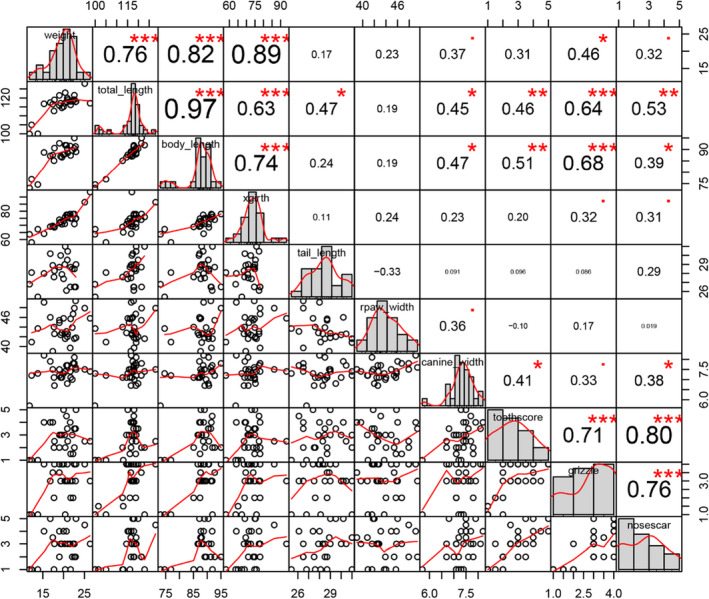
Correlation matrix (Peterson et al., 2018) of known‐age wild female (*n* = 32) morphometrics and life stage indices. Morphometrics examined are weight (kg), total length (cm, straight dorsal measure from nose to tail tip), body length (cm, total length minus tail length), xgirth (cm, transverse circumference at xyphoid or sternal process), tail length (cm), rpaw width (cm, right paw width), and canine width (mm). Life stage indices are tooth score, or degree of overall dental attrition from wearing, pitting, and fracturing as (1) none, (2) slight, (3) moderate, (4) severe, and (5) extreme; grizzle or pelage pigmentation loss as (1) none, (2) to eyes, (3) to lambdoidal crest, (4) to chest, and (5) to tail; and nose scarring as (1) none/black, (2) slight or white, (3) moderate or pink, (4) severe or red, and (5) extreme or significant tissue damage and loss

Using these metrics, the model that best predicted female known‐age included total length, and indices for nose scar and teeth condition. This model explained more than 94% of observed variance, confirming that teeth condition has the greatest model importance (42.7%). Nose scar index was second at 33.0%, followed by total length at 24.3%. Among our sample of known‐age females, however, nose scar and teeth condition indices were highly correlated (Figure B3). A simpler model using only teeth condition and total length (Figure 3) still explained more that 93% of observed variance, with relative importance of teeth condition and length at 66.1% and 33.9%, respectively:

(2) $$\ln\left(\text{female age}\right)=1.21*\ln\left(\text{toothscore}\right)+0.039*\text{total length}-3.76$$

Repeated k‐fold cross‐validation techniques yielded mean prediction error rates <1.2 years with model correctly assigning age within 1 and 2 years at 56% and 86%, respectively. With a limited sample of 14 known‐age males, a model derived from teeth condition scores and length (Figure 3) explained 98% of observed variance with respect to age:

(3) $$\ln\left(\text{male age}\right)=1.24*\ln\left(\text{toothscore}\right)+0.016*\text{total length}-1.42$$

Again, teeth condition had a high relative importance (71.0%), followed by total length (29.0%). When testing reliability of standard field aging methods, these estimates closely approximated model predictions for both females and males, with sample correlations *r* of 0.90 and 0.89, respectively.

**FIGURE 4:**
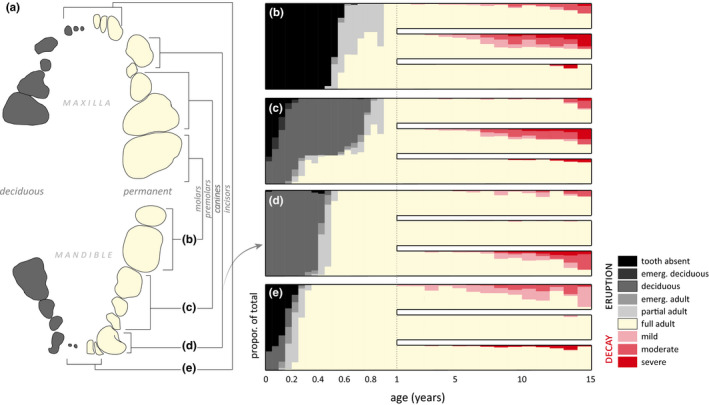
Sea otter dentition from first eruption to final decay. (a) Deciduous and permanent tooth diagrams, accompanied by charts of (b) molar, (c) premolar, (d) canine, and (e) incisor eruption and decay. From age 0–1, eruption is charted in 2‐week increments from 38 individuals of known ages. This is across six functional stages moving from absent (black) to fully emerged adult teeth (ivory). From age 1–15, we describe the proportion of adult teeth with wearing, pitting, and fracturing from 219 sea otters. The intensity of tooth decay is symbolized with increasing red saturation. Tooth function senesces almost completely by 15 years, significantly limiting foraging and survival

By characterizing incisor, canine, premolar, and molar eruption and decay timelines, we investigated the lifespan of each of these broad functional tooth categories and identified their relative vulnerability to attrition from wearing, pitting, and fracturing during aging (Figure 4). Type and degree of attrition varied by tooth category. Incisors were most vulnerable to wearing, molars and premolars were more susceptible to pitting, and canines fractured most frequently. Overall tooth lifespan was variable, measuring approximately 13–17 years, but signs of attrition among all types began as early as age 3 with some individuals experiencing significant declines in tooth condition by age 7–8. One known‐age female had no remaining functional teeth by age 9 and died in the wild from starvation. Others, by contrast, survived in the wild past 15 years of age with every tooth manifesting evidence of attrition.

**FIGURE 5:**
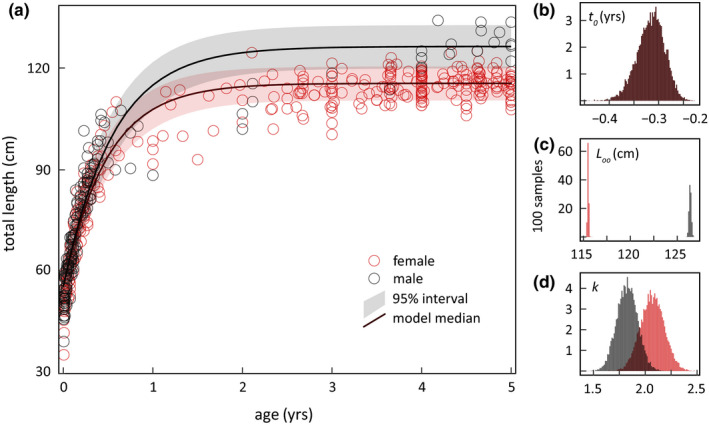
A derived Von Bertalanffy (VBGF) model for the age–length relationship of wild sea otters. (a) Mean model fit and prediction interval of total length at age from 761 otters (274 males, 487 females). This model is fit to all three VBGF parameters, (b) *t*_0_, (c) *L_oo_*, and (d) *k,* and run 10,000 times where each run randomly selects the age from an estimated distribution for that individual otter (see Methods). Although the majority of individuals (*n* = 523, 68.7%) was examined during a long‐term population monitoring study, a large sample of pups in this analysis (*n* = 238, 31.3%) are from live‐stranded individuals, some reared in captivity for release (*n* = 57). For animals with repeated health examinations and age–length records, each model run randomly draws a single length at age for each individual. To focus on the growth record period, we truncate the *x*‐axis to 5 years of age

From total length at age relationships for wild sea otters (Figure 5a), our mean VBGF estimate for −*t*_0_ was 115 days (range 83–163 days, Figure 5b). Mean asymptotic total length for males and females was 126.4 cm (126.0–126.8 cm) and 115.5 cm (115.3–115.7 cm, Figure 5c), respectively, with samples ranging from 118.3 to 138.0 cm for adult males and from 105.0 to 128.0 cm for adult females. Mean somatic growth, *k*, was significantly greater for females (2.1 yr^−1^, 1.6–2.5 yr^−1^) than males (1.8 yr^−1^, 1.4–2.2 yr^−1^, Figure 5d).

**FIGURE 6:**
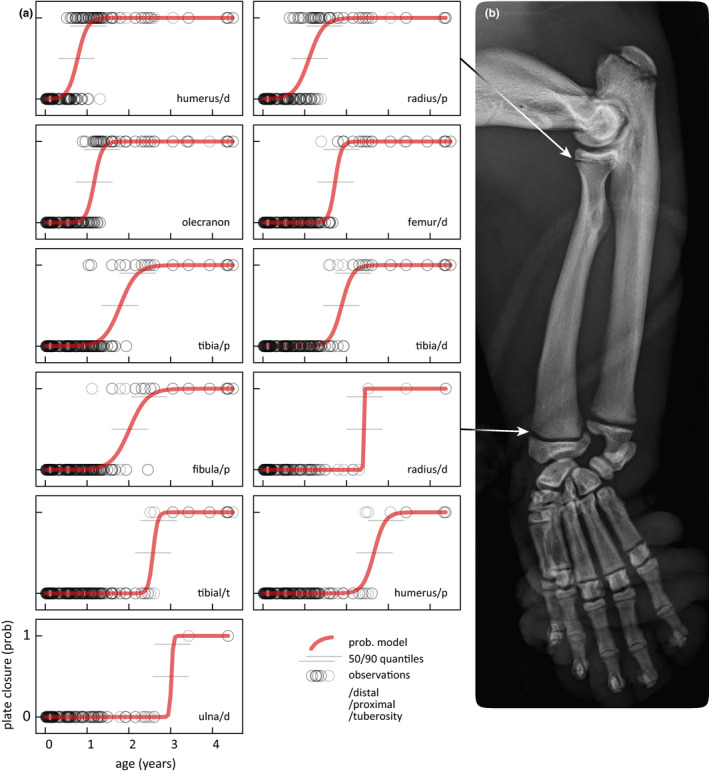
Fusion timelines of long‐bone epiphyseal plates may provide a reliable method to age sea otters from 1 to 3 years old. (a) Probability of closure for eleven growth plates by age is estimated using binomial logistic regression models (red line) fitted to empirical observations of plate status (either “open” or “closed”) from radiographs (open black circles) from 37 known‐age individuals, <4 years old. (b) Forearm radiograph of a juvenile male (age ~ 10 months) with arrows indicating open proximal and distal epiphyseal plates of the radius. Future studies may further refine the accuracy of aging techniques with additional derived morphometric relationships to age, such as shown here with epiphyseal plate closures

Closure of long‐bone epiphyseal plates span throughout late juvenile and subadult stages (0.7–3 years of age), with identifiable metrics during each year of development (Figure 6). The distal humerus within the forelimb near the elbow is the first growth plate to close at approximately 0.77 years (1.0 year, 90% quartile). Other growth plates within the elbow joint (i.e., proximal radius and olecranon) fuse around 1 year of age, at 1.1 year (1.5 years, 90% quartile) and 1.2 years (1.4 years, 90% quartile). Closer to 2 years of age, long bones in the hind limb near the knee, shin, and ankle (i.e., distal femur, proximal and distal tibia, and proximal fibula) begin to close. At approximately 2.5 years of age, growth plates among an assortment of joints including wrist (distal radius), knee (tibial tuberosity), and shoulder (proximal humerus) fuse. The distal ulna in the wrist or forelimb was the last to close at approximately 3 years of age. Among growth plate closures through age 1.5 years (distal humerus, proximal radius, and olecranon), when radiographs from both males and females were available, female growth plates closed 4 to 5 months earlier than for males.

## 4 DISCUSSION

By synthesizing more than two decades of physical examination data from strandings, captive‐rearing for release, and field studies, we identify five key findings related to aging wild southern sea otters and inform standard field aging methods. First, tooth eruptions and replacements follow a predictable progression from week two to approximately 9 months of age, when permanent dentition is complete (Figure 2, Tables A1 and A2). Second, teeth condition is the single best predictor of age, and when combined with total length explained more than 93% of variability related to aging (Figures 3 and 4). Third, from patterns of tooth attrition, lifespan of sea otter dentition is approximately 13–17 years, consistent with maximum life expectancy (Figure 4). Fourth, VBGF models applied to length at age measurements yielded biologically relevant estimates for population parameters, including gestation at approximately 17 weeks (Figure 5). Fifth, radiographs of long‐bone growth plates may increase accuracy of aging sea otters between juvenile and adult stages (Figure 6), enhancing our ability to characterize this important life history transition.

Tooth eruption and replacement schedules derived from known‐age captive pups provided a reliable and detailed reference for aging wild sea otters. This finding is similar among aging studies of other mammalian species (Binder & Van Valkenburgh, 2000; Kahumbu & Eley, 1991; Lee, Sayialel, Lindsay, & Moss, 2012; Olifiers, de Cassia Bianchi, D'Andrea, Mourao, & Gompper, 2010; Stander, 1997; Van Horn et al., 2003). When accurate, eruption schedules are consistent within a single species distributed across a broad or extensive geographic range or even between subspecies (Haynes, 1984; Kahumbu & Eley, 1991; Miller, 1972). For this reason, an earlier subset of tooth stage‐by‐age observations of southern sea otter pups serves as a proxy to refine aging criteria of northern sea otters in Washington, where similar information is unavailable (Schuler et al., 2018). We refine and boost accuracy of these findings by restricting analysis to pups stranding as newborns (age 2 weeks or fewer), and nearly doubling sample size (21 vs. 38). Within California, this metric is likely applicable across the population's extent, where all sea otters descended from a remnant colony that survived commercial hunting and still exhibit low overall genetic diversity (Beichman et al., 2019; Larson, Jameson, Etnier, Fleming, & Bentzen, 2002; Larson, Jameson, Etnier, Jones, & Hall, 2012). Nutritional status may also influence tooth eruption timelines (Kahumbu & Eley, 1991; Phillips‐Conroy & Jolly, 1988) but its effect is presumably less than with other metrics, such as weight or size. This is evident from strandings of chronically undernourished and undersized juvenile sea otters, resembling pups in size only, but demonstrating advanced prey handling skills and a full set of permanent teeth.

Among all features related to external morphology, the single best predictor of age after juvenile stage (≥1 year) was tooth attrition, which is consistent with other minimally invasive aging studies using known‐age individuals (Chevallier et al., 2017; Delahay et al., 2011; Galbany et al., 2018; Gipson et al., 2000; Stander, 1997). Wild sea otters are primarily susceptible to dental attrition from foraging on hard‐shelled invertebrates (Fisher, 1941; Kenyon, 1969; Winer, Liong, & Verstraete, 2013), many which burrow in sand. Shells and sand may directly crack, fracture, or wear the teeth by shortening the distance from tip to gumline (crown). When tooth enamel cracks, grains of sand also may invade and abrade the underlying soft dentine, undermining the tooth and creating deep, cavernous pits much greater than the original sand particles (Kenyon, 1969; Lawn, Lee, Constantino, & Lucas, 2009). Nose scar index was another notable female aging metric. This feature, however, may be less relevant for aging females outside the range center where competition for resources is less acute (Tinker et al., 2017), and strandings due to mating trauma are rare (Nicholson et al., 2018). Interestingly, nose scarring was also highly correlated with tooth condition (Figure B3), perhaps indicating that mating trauma may be aggravated as females lose their primary means of defense against aggressive suitors. Grizzle, which is useful in aging northern sea otters (Garshelis, 1984), was not a reliable predictor of female age. Paw width, tail length, and canine width by comparison were all highly unreliable for age estimation. Ultimately, sea otter aging models derived from teeth condition scores and total length successfully predicted age at a greater rate than reported by cementum aging studies (Bodkin et al., 1997), indicating that acceptable age estimates of wild individuals may be achieved from two noninvasive metrics.

The overall trend in tooth eruption and attrition through time is collectively informative. Dental attrition is universally present as sea otters age and may be a significant factor affecting long‐term survival, especially after age 8–9 years. Although we found a clear correlation between age and tooth wear, we also suspect variation in dental condition related to gender (Marti & Ryser‐Degiorgis, 2018; Stander, 1997). Males spar when defending territories, by chasing, pouncing, and biting. These aggressive encounters may increase incidence of tooth fractures, especially to canines, which compounds wear on overall dentition through time. From our few known‐age wild males, maximum lifespan was approximately 13–15 years when dentition was marked by dramatic tooth fractures and loss. By contrast, wild females overall experienced less severe tooth attrition until a few years later, extending their maximum lifespan to age 15–17 years.

Dental attrition was more variable with age and described slightly less model variation among females than males (66% vs. 71%). This result may be explained by differences between male and female life histories. Across their lifespan, females tend to be less vagile (Tarjan & Tinker, 2016) and likely more influenced by local conditions than males. For this reason, we expect that female dental attrition patterns may also be explained by their individual diet (Tinker et al., 2012), foraging habitat, and tool use frequency (Fujii, Ralls, & Tinker, 2017), in addition to age, but more analyses are required to address these specific questions. With our modest sample, females foraging predominantly in harbors or estuaries, where infaunal clams and worms are common (Tinker et al., 2018), experienced minimal molar wearing or pitting with age compared with their counterparts occupying open coastal kelp forests, where mussels, crabs, and urchins comprise the majority of the population's diet (Tinker et al., 2017).

By drawing on thirty years of pup strandings, we enhanced our sample of wild individuals to represent every life stage, including infancy. This allowed us to build on previous growth model approaches typically sparse in data from ages 0 to 1 year (Laidre et al., 2006; Monson, 2009; Tinker et al., 2013, 2018), and develop a more demographically and empirically representative model across the entire life history of the species. With a more rigorous VBGF fit, we were able to explore model reliability of estimating duration of active gestation. Females mate directly after weaning their pup, then experience delayed implantation when the resulting blastocyst enters a rest phase before implantation and embryo formation (Kenyon, 1969; Riedman & Estes, 1990). Without obvious external cues, active gestation or the phase after implantation and before birth is difficult to measure. Theoretical and hormonally derived mean gestation estimates for northern sea otters range from approximately 107 to 117 days (Huggett & Widdas, 1951; Larson, Casson, & Wasser, 2003), consistent with our mean model estimate of 115 days. Somatic growth estimates were also comparable with similarly sized species (Palomares & Pauly, 2008). Male and female mean asymptotic lengths resembled estimates derived from sea otters captured along mainland California from 1995 to 2000 (Laidre et al., 2006), but midway between estimates from Alaskan sea otter populations when at carrying capacity and during steep decline (Laidre et al., 2006).

Long‐bone growth plate fusion timelines are a potentially reliable resource for assessing age and maturity of sea otters throughout the year 2–3 transition from juvenile to adulthood (Gandal, 1954; Zeiler, 1988). Within our sample, all males were released to the wild by age 1.5 years or transferred to another captive facility, so we only examined females to adulthood. Even with limited male samples, we detected fusion rate differences between males and females among early closing plates within the forelimb (distal humerus, proximal radius, and olecranon). Earlier plate closure in females is common among sexually dimorphic mammals (Flinn et al., 2013; Malina & Bouchard, 1991; Marks & Erickson, 1966; Purdue, 1983). It also corresponds with our findings that female sea otters mature faster (Figure 5d) than males. In addition to gender, a captive setting may also affect growth plate closure rates by optimizing nutritional health. Captivity has been shown to both delay and accelerate the timing of fusion depending on study (Flinn et al., 2013), so more work is needed to better understand underlying mechanisms.

As our collective interest in mammalian wildlife has evolved from harvest to protection, novel and noninvasive aging techniques are emerging to assess living populations (Chevallier et al., 2017; Delahay et al., 2011; Marti & Ryser‐Degiorgis, 2018; Olifiers et al., 2010). For southern sea otters, traditionally accepted cementum annuli aging is sparsely tested and validated, often unpublished, skewed toward immature individuals (Siniff & Ralls, 1988), or combined with samples from northern sea otters in Alaska where distinct seasonality is advantageous relative to California (Bodkin et al., 1997). After decades of field research, these narrow findings reflect this aging method's limitations when applied to free‐ranging sea otters in California. Tooth extraction also may be costly to a living carnivore that relies heavily on its dentition for survival, especially as it ages. As an effective and conservation‐minded alternative, we provide strong support for aging techniques derived from less invasive methods.

In the absence of reliable cementum annuli aging during the last two decades, we gathered data to ground truth standard field aging techniques and design a model to reduce errors from observer bias, inexperience, and fatigue. Our model strongly validates the use of teeth condition indices, while dismissing other metrics (e.g., grizzle) often considered during field aging (Tinker et al., 2013, 2017, 2018). We derived this model from known‐age sea otters primarily occupying the center of the range, both open coast and estuary habitats, so it may not reliably represent individuals from more extreme frontiers where both diet and genetic isolation could significantly alter aging patterns. This includes translocated or reintroduced populations exposed to novel prey resources or vulnerable to foundering effects from an extremely small source size (e.g., San Nicolas Island; Hatfield, 2005).

Sea otter recovery efforts during the last thirty years have provided a unique opportunity to retrospectively examine individual morphometrics and life history indices in the context of aging. During this time, we have recorded information representing every sea otter life stage throughout the entire mainland California range by combining information from strandings, especially among pups, with long‐term field studies. Our results highlight the value of gathering repeated observations of individuals through time, not only in the wild but also in captivity. Captive facilities (zoos, aquariums, wildlife sanctuaries, and rehabilitation centers) provide a resource where animals may be examined thoroughly, frequently, and humanely to document morphological development with age. In combination with longitudinal studies of known‐age individuals in the wild, this allowed us to ground truth standard field aging methods and determine best metrics available to describe how sea otter morphology changes with age. These minimally invasive aging techniques provide the means to gather age‐specific information from a living wild population to inform management decisions focused on restoring sea otters and their ecosystems throughout California.

## CONFLICT OF INTEREST

The authors declare no competing interests.

## AUTHOR CONTRIBUTIONS

**Teri E. Nicholson:** Conceptualization (equal); data curation (equal); formal analysis (equal); investigation (equal); methodology (equal); project administration (equal); visualization (supporting); writing – original draft (lead); writing – review & editing (lead). **Karl A. Mayer:** Data curation (equal); investigation (equal); methodology (equal); writing – review & editing (supporting). **Michelle M. Staedler:** Data curation (equal); investigation (equal); methodology (equal); writing – review & editing (supporting). **Tyler O. Gagne:** Formal analysis (equal); visualization (equal); writing – original draft (supporting); writing – review & editing (supporting). **Michael J. Murray:** Data curation (supporting); methodology (supporting); writing – original draft (supporting); writing – review & editing (supporting). **Marissa A. Young:** Data curation (supporting); methodology (supporting); writing – review & editing (supporting). **Joseph A. Tomoleoni:** Funding acquisition (equal); investigation (supporting); resources (equal); writing – original draft (supporting); writing – review & editing (supporting). **M. Tim Tinker:** Funding acquisition (equal); investigation (supporting); methodology (supporting); resources (equal); writing – original draft (supporting); writing – review & editing (supporting). **Kyle S. Van Houtan:** Conceptualization (equal); formal analysis (equal); funding acquisition (equal); project administration (lead); resources (equal); supervision (lead); visualization (lead); writing – original draft (supporting); writing – review & editing (supporting).

## ETHICAL APPROVAL

Our study operated under USGS permit #MA672624‐18, U.S. Fish & Wildlife Service permits #MA032027‐2 and LOA032027‐2, and Monterey Bay Aquarium permits #MA186914 and #032027. All animal handling was in accordance with the requirements of USDA Class R Research Facility license # 93‐R‐0476. Any use of trade, firm, or product names is for descriptive purposes only and does not imply endorsement by the U.S. Government.

## Data Availability

All the raw data used in this project are available at a third party repository (https://osf.io/qyjdc).

## Appendix A

### Tooth first and full eruption timelines

**TABLE A1:** Age (median, minimum, 2.5%, 25%, 75%, 97.5%, and maximum; in days) of first eruption for four deciduous (lower case) and 16 permanent (uppercase) sea otter teeth

Tooth	Median	Min	2.5%	25%	75%	97.5%	Max	# otters
premolar_3_	9	3	4	6	13	18	19	21
premolar^3^	19	9	9	17	24	32	33	30
premolar_4_	24	9	9	17	28	32	32	35
INCISOR^1^	31	12	16	23	35	44	50	35
premolar^4^	38	28	28	33	43	55	56	31
INCISOR_1_	39	26	27	34	42	51	54	32
INCISOR^2^	41	28	28	35	43	55	61	31
PREMOLAR^2^	50	36	37	47	60	69	70	22
INCISOR_2_	54	38	39	49	59	65	66	24
INCISOR^3^	55	38	40	49	61	71	74	24
PREMOLAR_2_	56	36	39	47	61	69	70	22
CANINE_1_	104	82	82	100	111	120	124	19
CANINE^1^	106	83	88	101	111	116	117	15
MOLAR^1^	133	112	114	127	147	164	169	11
MOLAR_1_	139	123	124	128	147	162	166	9
MOLAR_2_	152	126	126	147	167	169	169	9
PREMOLAR_3_	179	146	148	164	190	201	203	12
PREMOLAR^3^	187	146	150	174	202	205	205	8
PREMOLAR^4^	202	146	153	199	204	206	206	7
PREMOLAR_4_	205	197	197	201	206	206	206	3

Note

Mandibular teeth are represented by subscript, and maxillary are indicated by superscript. Subscript or superscript number corresponds with tooth order or position relative to front or center. Sample size or number of individuals is listed as # otters. Eruption timelines related to sidedness (left versus. right; *t* = 1.29, *p* = .21) and sex (males vs. females; *t* = 0.64, *p* = .53) were similar, so we pooled data based on these two factors.

**TABLE A2:** Age (median, minimum, 2.5%, 25%, 75%, 97.5%, and maximum; in days) of full emergence for four deciduous (lowercase) and 16 permanent (uppercase) sea otter teeth

Tooth	Median	Min	2.5%	25%	75%	97.5%	Max	# otters
premolar_3_	37	9	17	31	43	58	64	34
premolar^3^	49	32	34	43	59	73	81	31
premolar_4_	46	30	31	40	55	67	70	31
INCISOR^1^	64	46	48	58	69	83	88	28
premolar^4^	74	46	54	66	79	89	93	27
INCISOR_1_	63	46	48	56	70	98	102	28
INCISOR^2^	65	46	50	59	70	83	88	27
PREMOLAR^2^	76	47	52	66	83	106	116	29
INCISOR_2_	68	46	51	62	76	91	97	28
INCISOR^3^	76	46	54	69	82	89	93	27
PREMOLAR_2_	76	47	52	66	83	112	116	29
CANINE_1_	133	102	106	126	146	162	169	13
CANINE^1^	133	102	108	128	146	162	169	13
MOLAR^1^	182	146	148	165	216	259	264	11
MOLAR_1_	176	146	146	167	189	210	210	13
MOLAR_2_	191	146	146	169	205	225	228	12
PREMOLAR_3_	205	164	169	196	214	249	256	8
PREMOLAR^3^	224	214	214	215	238	254	256	4
PREMOLAR^4^	216	212	212	214	243	255	256	5
PREMOLAR_4_	243	214	215	228	249	255	256	3

Note

Mandibular teeth are represented by subscript, and maxillary are indicated by superscript. Subscript or superscript number corresponds with tooth order or position relative to front or center. Sample size or number of individuals is listed as # otters. Eruption timelines related to sidedness (left versus. right; *t* = 0.65, *p* = .52) and sex (males vs. female; *t* = 0.40, *p* = .69) were similar, so we pooled data based on these two factors.
